# A 61-year-old Female with Right Upper Abdominal Pain

**DOI:** 10.5811/cpcem.2020.7.48514

**Published:** 2020-09-17

**Authors:** Drew A. Long, Brit Long

**Affiliations:** Brooke Army Medical Center, Department of Emergency Medicine, Fort Sam Houston, Texas

**Keywords:** cholecystitis, cholecystectomy

## Abstract

**Case Presentation:**

A 61-year-old female presented to the emergency department with right upper quadrant abdominal pain following a cholecystectomy 18 days prior. Computed tomography (CT) of her abdomen demonstrated a large abscess in her post-hepatic fossa. She was admitted to the general surgery service and received an image-guided percutaneous drain placement with interventional radiology with immediate return of purulent material. She was discharged home after a three-day hospital course with outpatient antibiotics and follow-up.

**Discussion:**

Patients may have multiple complications following cholecystectomy, including infection, bleeding, biliary injury, bowel injury, or dropped stone. The emergency clinician must consider cholecystectomy complications including gallbladder fossa abscess in patients presenting with abdominal pain in the days to weeks following cholecystectomy, especially if they present with signs of sepsis. Critical actions include obtaining CT and/or ultrasonography, initiating broad spectrum antibiotics, and obtaining definitive source control by either surgery or interventional radiology.

## CASE PRESENTATION

**Image 1:**
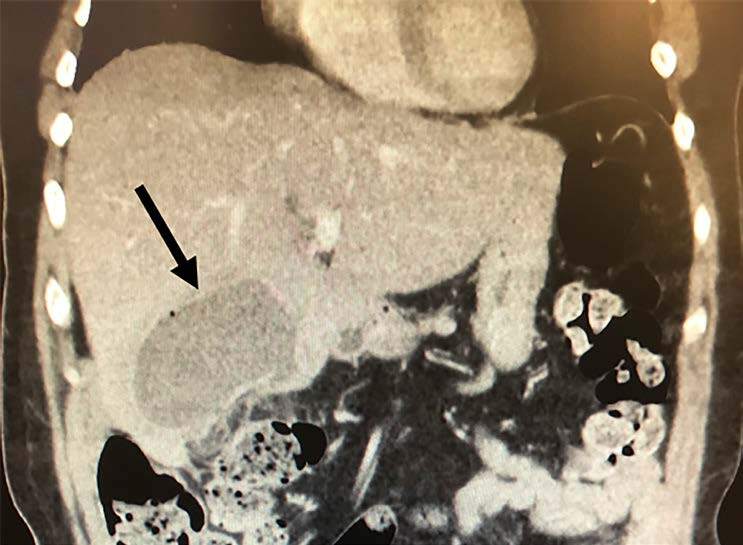
Sagittal view of computed tomography of abdomen/ pelvis with intravenous contrast demonstrating a 7.3 × 5.7 × 7.7 centimeter rim-enhancing fluid collection in the gallbladder fossa, denoted by black arrow.

**Image 2:**
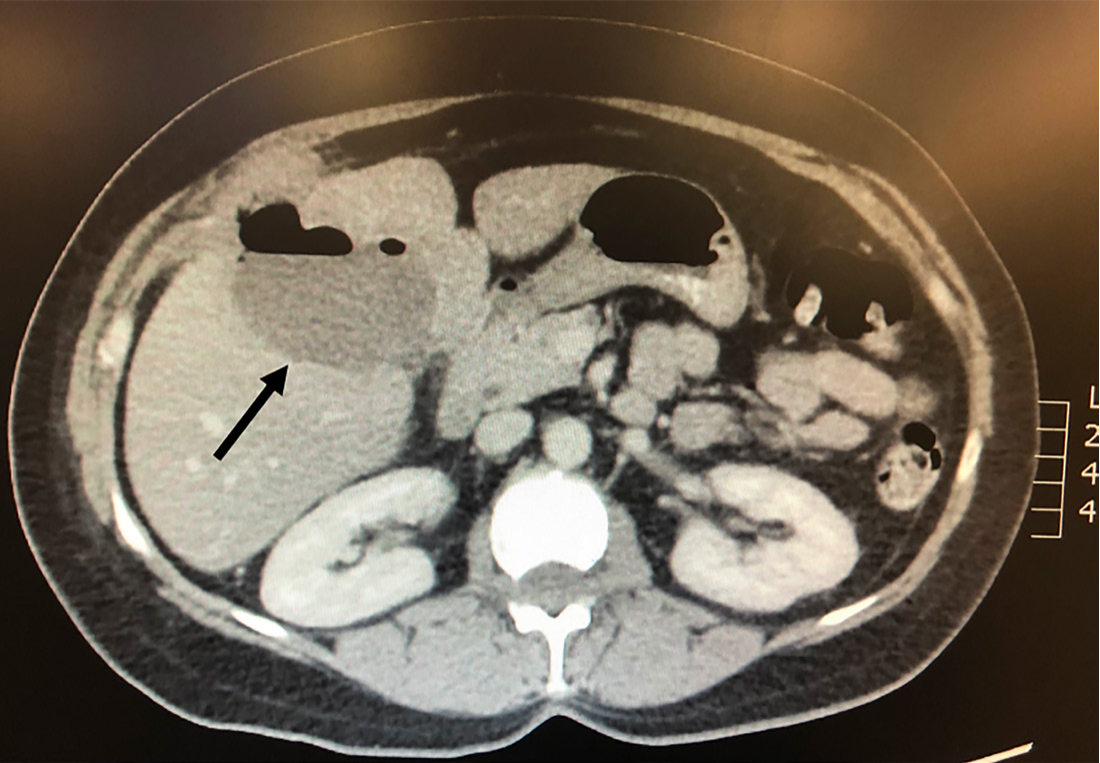
Transverse view of computed tomography of abdomen/pelvis with intravenous contrast demonstrating a 7.3 × 5.7 × 7.7 centimeter rim-enhancing fluid collection with air-fluid level in the gallbladder fossa, denoted by black arrow.

A 61-year-old female presented to the emergency department with right upper quadrant (RUQ) abdominal pain for the prior several days. She had a laparoscopic cholecystectomy 18 days prior to presentation for operative management of cholecystitis. Initial vital signs were notable for a heart rate of 110 beats per minute. Abdominal examination demonstrated moderate RUQ abdominal tenderness to palpation. Laboratory evaluation revealed a white blood cell count of 11.3 per microliter (μL) (normal range 4.5–11 μL), total bilirubin 0.5 milligram per deciliter (mg/dL) (normal <1.2 mg/dL), aspartate aminotransferase of 30 international units per liter (IU/ L) (normal 5–40 IU/L), alanine aminotransferase of 45 IU/L (normal 7–56 IU/L), and alkaline phosphatase of 247 IU/L (normal 20–140 IU/L). Computed tomography (CT) with intravenous (IV) contrast of her abdomen and pelvis demonstrated a 7.3 × 5.7 × 7.7 centimeter rim-enhancing fluid collection with air-fluid level in the gallbladder fossa (Images 1 and 2).

## DISCUSSION

The patient presentation and imaging were concerning for gallbladder fossa abscess, and she received 4.5 grams of piperacillin/tazobactam IV in addition to 2 L of IV crystalloid and was admitted to the surgical service. Interventional radiology placed an image-guided drain, with 100 milliliters of purulent material expressed upon placement. The patient was discharged home on hospital day three with the drain in place on antibiotics with follow-up.

Cholecystectomies are commonly performed in the United States, most commonly laparoscopically. Potential complications include bile duct injury, biliary stricture, bowel injury, bleeding, infection, and dropped gallstones.^1^,^2^ Complications, particularly infection, are more common in patients with underlying immunosuppression such as diabetes.^3^ In patients presenting with abdominal pain after cholecystectomy, these complications should be considered and imaging with either CT or ultrasonography obtained. If a patient has signs of sepsis (eg, tachycardia, fever) days to weeks following cholecystectomy, surgical site infection or abscess formation must be considered and appropriate antibiotic treatment with resuscitation provided. Definitive management of a gallbladder fossa abscess is source control with operative drainage or drain placement.^4^

CPC-EM Capsule

What do we already know about this clinical entity?

*Patients may have multiple complications following cholecystectomy, including biliary and bowel injuries, bleeding, dropped gallstones, and infection*.

What is the major impact of the image(s)?

*This image depicts a gallbladder fossa abscess, an uncommon complication following cholecystectomy*.

How might this improve emergency medicine practice?

*Gallbladder fossa abscess should be suspected in patients presenting with abdominal pain or signs of sepsis in the days to weeks following cholecystectomy*.
